# Identification of Potential Antigens for Developing mRNA Vaccine for Immunologically Cold Mesothelioma

**DOI:** 10.3389/fcell.2022.879278

**Published:** 2022-07-01

**Authors:** Shichao Zhang, Shuqin Li, Ya Wei, Yu Xiong, Qin Liu, Zuquan Hu, Zhu Zeng, Fuzhou Tang, Yan Ouyang

**Affiliations:** ^1^ Key Laboratory of Infectious Immune and Antibody Engineering in Guizhou Province, School of Biology and Engineering, Guizhou Medical University, Guiyang, China; ^2^ Immune Cells and Antibody Engineering Research Center of Guizhou Province, School of Biology and Engineering, Guizhou Medical University, Guiyang, China

**Keywords:** mesothelioma, tumor antigen, immune subtype, mRNA vaccine, immunogenomic landscape

## Abstract

Messenger RNA vaccines are considered to be a promising strategy in cancer immunotherapy, while their application on mesothelioma is still largely uncharacterized. This study aimed to identify potential antigens in mesothelioma for anti-mesothelioma mRNA vaccine development, and further determine the immune subtypes of mesothelioma for selection of suitable candidates from an extremely heterogeneous population. Gene expression data and corresponding clinicopathological information were obtained from the TCGA and gene expression omnibus, respectively. Then, the genetic alterations were compared and visualized using cBioPortal, and differentially expressed genes and their prognostic signatures were identified by GEPIA. The relationship between tumor-infiltrating immune cells and the expression of tumor antigens was systematically evaluated by TIMER online. Finally, the immune subtypes and immune landscape of mesothelioma were separately analyzed using consensus cluster and graph learning-based dimensional reduction. A total of five potential tumor antigens correlated with prognosis and infiltration of antigen-presenting cells, including AUNIP, FANCI, LASP1, PSMD8, and XPO5 were identified. Based on the expression of immune-related genes, patients with mesothelioma were divided into two immune subtypes (IS1 and IS2). Each subtype exhibited differential molecular, cellular and clinical properties. Patients with the IS1 subtype were characterized by an immune “cold” phenotype, displaying superior survival outcomes, whereas those with the IS2 subtype were characterized by an immune “hot” and immunosuppressive phenotype. Furthermore, immune checkpoints and immunogenic cell death modulators were differentially expressed between the IS1 and IS2 immune subtype tumors. The immunogenomic landscape of mesothelioma revealed a complex tumor immune microenvironment between individual patients. AUNIP, FANCI, LASP1, PSMD8, and XPO5 are putative antigens for the development of anti-mesothelioma mRNA vaccine and patients with the IS1 subtype may be considered for vaccination.

## Introduction

Malignant mesothelioma is one of the most devastating and aggressive solid tumors, with less than a 10% survival rate at 5 years (Huang et al., 2019). Worldwide, there were 30,870 and 26,278 estimated new mesothelioma cases and deaths, respectively, in 2020 (Sung et al., 2021). Although it is not a common tumor type, the majority of mesothelioma patients are diagnosed at an advanced stage or metastatic phase due to the absence of early symptoms (Fels Elliott and Jones, 2020). Accompanied by progress in medical technology, several therapeutic approaches, such as surgery, radiation, chemotherapy and combination treatment extend the survival of mesothelioma patients. However, the current outcome of treatment remains unsatisfactory (Taioli et al., 2015; Fels Elliott and Jones, 2020; Viscardi et al., 2020). Therefore, novel treatment strategies are urgently needed to improve the outcome of patients with mesothelioma.

Innate and adaptive immunity play key roles in the initiation, progression, and prognosis of tumors. In the three phases of tumor immunoediting, the host immune system can eliminate tumor cells or condition tumor immunogenicity to promote disease progression. Immunotherapy based on immune checkpoint (ICP) inhibitors (e.g., PD-1/PD-L1 and CTLA-4) has achieved success in combating some malignant tumors, however, only a minority of patients with mesothelioma benefit from ICP inhibitor therapy (Chatwal and Tanvetyanon, 2018; Mutti et al., 2018; Forde et al., 2019). After targeting ICP proteins, tumor vaccines are considered to be another effective tool for immunotherapy (Chiang et al., 2010; Wang et al., 2018). This has become attractive to oncologists because vaccines can promote anti-tumor-specific T cell responses by inducing active immunity. Recently, a CTGF/MSLN tumor vaccine targeting MSLN-expressing malignant mesothelioma generated anti-tumor effects by decreasing tumor volumes and prolonging survival (Chen et al., 2018a). Also, an induced pluripotent stem cells (iPSC) vaccine suppressed mesothelioma progression by promoting tumor antigen-specific T cell responses (Kooreman et al., 2018). Although the therapeutic effects of this clinical application remain unclear, evidence is sufficient to warrant further studies on the potential of mesothelioma-associated vaccines.

Tumor vaccines can trigger and enhance anti-tumor-specific immune responses by reprogramming the immune system to identify and eliminate malignant tumor cells (Chiang et al., 2010; Wang et al., 2018). They exhibit a broad therapeutic window, limited non-specific action, few adverse effects, and produce a long-term immunological memory response. Thus, tumor vaccine therapies have several advantages (e.g., less vulnerable to develop drug resistance and adverse reactions, and durable therapeutic effects) compared with standard chemotherapy and immunotherapy. Based on the antigen form, tumor vaccines can be classified into tumor cell, dendritic cell (DC), peptide, DNA, and RNA types (Mockey et al., 2007; Bouzid et al., 2020). However, the first four vaccine types have shortcomings limiting their potential for clinical application. For example, tumor cell vaccines have shown promising results in laboratory experiments and preclinical models, but have failed in clinical trials because of poor tumor cell immunogenicity (Emens, 2006; Chiang et al., 2010). Although DC vaccines loaded with tumor antigens can induce cytotoxic T cell and T helper cell responses, the effects are not satisfactory (Emens, 2006). Peptide-based tumor vaccines in clinical testing exhibit significantly enhanced immunogenicity and clinical anti-tumor activity. Unfortunately, malignant mesothelioma is not a candidate for surgical therapy, so it is difficult to obtain tumor genomic data for developing personalized peptide vaccines (Sayour et al., 2018; Viscardi et al., 2020). Moreover, the integration of DNA-encoded antigen sequences into the genome of tumor cells has mutation risks, leading to suboptimal treatment (Myhr, 2017; Pardi et al., 2018). Conversely, mRNA vaccines do not integrate into the genome or interact with DNA, thus they do not result in a mutation risk to the host. The half-life of mRNA *in vivo* may be modulated through various modifications or delivery systems. Also, the inherent immunogenicity of mRNA can be downregulated to ensure an acceptable safety profile (Pardi et al., 2018). Additionally, various modifications and carrier strategies can enhance mRNA stability, cellular uptake, and expression (McNamara et al., 2015; Sayour et al., 2015). Through *in vitro* transcription reactions, rapid development and production of mRNA vaccines may be achieved, which reduces the time needed to reach the clinic. It is easy to encode any pathological antigen by designing and modifying the mRNA sequence, which is important for constructing individualized mRNA vaccines (Luo et al., 2020). Therefore, in the context of clinical applications, mRNA vaccines may be the best choice for targeting tumor-specific antigens (TSAs). Preclinical models and clinical trials have indicated that mRNA vaccines encoding TSAs exhibit potent anti-tumor activity in multiple tumors, such as melanoma, gastrointestinal cancer, colorectal cancer, pancreatic adenocarcinoma, and hepatocellular carcinoma (Pardi et al., 2018; Pardi et al., 2020; Xu et al., 2020).

Although mRNA tumor vaccines have more advantages compared with other conventional vaccine platforms, it remains challenging to develop specific potent antigens from numerous mutated candidates. For patients with mesothelioma, no effective mRNA vaccine has been reported and the subpopulation that may benefit from an mRNA vaccination remains undefined. Tumor-infiltrating immune cells and immune-related genes (IRGs) play important roles in regulating anti-tumor immune responses and determining patient survival (Oehl et al., 2018; Fusco et al., 2020). Therefore, IRG-based immuno-subtyping can be exploited to stratify mesothelioma patients and select suitable candidates for mRNA vaccination.

In this study, five putative tumor antigens were identified for mesothelioma mRNA vaccine development. These tumor-associated antigens were associated with prognosis, genomic alterations, and antigen-presenting cell (APC) infiltration. Based on the expression of IRGs, we constructed a consistent cluster for identifying suitable mRNA vaccine candidates for mesothelioma patients. Two powerful immune subtypes (IS1 and IS2) were defined and validated, which exhibited different molecular, cellular and clinical characteristics. In addition, the immune landscape of individual patients was determined by graph learning-based dimensional reduction. Our findings provide valuable insight for the development and administration of mRNA vaccines for mesothelioma patients.

## Methods

### Data Acquisition

The workflow of this study is presented in Supplymentary Figure S1. The gene expression data and corresponding clinicopathological information of mesothelioma patients were obtained from The Cancer Genome Atlas (TCGA, https://www.cancer.gov/tcga; the discovery cohort; 86 cases) and Gene Expression Omnibus (GEO, https://www.ncbi.nlm.nih.gov/geo; the validation cohort; GSE163720, 131 cases) databases. Clinical information was collected on gender, age, clinical stage, grade, T-stage, and overall survival (OS). According to previous research, 2,108 immune-related genes were extracted for further analysis, including interleukins and interleukin receptors, chemokines and chemokine reports, cytokines and cytokine receptors, interferons, interferons receptors, TGF-β family members, antigen processing and presentation, TNF family members, and other immune-related genes.

### Data Pre-processing

The microarray expression data from GEO were pre-processed and aggregated, and the microarray probes with null gene detection values were excluded. Among the 22,148 expressed genes, 782 immune cell-related genes were retained. In TCGA tumour samples, those with incomplete clinical information were removed. Tumor patients with survival times of less than 90 days were further excluded from analysis. Furthermore, genes with an average value of Fragments Per Kilobase of exon model per Million mapped fragments (FPKM) < 0.5 were also eliminated.

### Gene Expression Profiling Interactive Analysis

Gene Expression Profiling Interactive Analysis (GEPIA, http://gepia2.cancer-pku.cn), which is an online tool (Tang et al., 2019), was used to evaluate the prognosis values of selected antigens. A 50% (median) cut-off value was set to evaluate the mesothelioma patients’ (overall survival) OS and relapse-free survival (RFS) in the Kaplan-Meier method, and the statistically significant differences between high- and low-expression groups were identified by the log rank test (*p* < 0.05).

### Genome Alteration Analysis

Based on the raw RNA-seq data, somatic mutation and copy number alterations in mesothelioma tumours were evaluated by the cBio Cancer Genomics Portal (cBioPortal, http://www.cbioportal.org) (Cerami et al., 2012). *p* < 0.05 was considered statistically significant.

### Immune Cell Infiltration Analysis

In the tumour microenvironment, the relationship between the infiltration abundance of immune cells and the expression of mesothelioma-associated genes was analyzed using the Tumour Immune Estimation Resource (TIMER, https://cistrome.shinyapps.io/timer/) (Li et al., 2017). *p* < 0.05 was considered statistically significant.

### Identification of immune Subtypes

According to the expression profiles of 2,108 immune-related genes, cluster analysis was performed by building the consistency matrix to detect the different gene modules and corresponding immune subtypes. In the partition around medoids (PAM) algorithm, the distance metric was determined by the 1-Pearson correlation coefficient with 500 bootstraps (each bootstrap included 80% mesothelioma samples). Based on the consensus matrix and consensus cumulative distribution function, the optimum partition of cluster sets (2–10) was selected to confirm the immune subtypes, which was verified by the gene expression omnibus (GEO) cohort. Furthermore, Pearson correlation and intra-group proportion were calculated to quantify the consistency of immune subtypes in the two cohorts (discovery and validation cohorts).

### Immune-Associated Signaling Pathways

The “clusterProfiler” package was used for gene oncology (GO) enrichment analysis (biological processes; BP) to explore the pathways of immune-associated molecular and cellular characteristics. The correlation between 56 immune-associated molecular characteristics and immune subtypes was evaluated, and the composition of immune cells in tumour tissues was analyzed using the CIBERSORT algorithm (Chen et al., 2018b).

### Gene Co-Expression Network Analysis

The immune-related gene co-expression modules were determined using the R package “WGCNA” (Yu et al., 2012), and the immunoenrichment score for each sample to measure the coordinated up- or downregulation of genes within a sample was calculated by single-sample Gene Set Enrichment Analysis (SSGSEA).

Correlation analysis between categories and some associated biological processes was conducted. According to previous reports, gene sets including several gene-related biological processes were constructed, and these biological pathways included antigen processing and presentation, DNA damage repair, pan-fibroblast TGF-β response signature (pan-F-TBRS), angiogenesis, DNA replication, nucleotide excision repair, mismatch repair, WNT targets, cell cycle, CD8^+^ T effectors, antigen processing machinery, and immune-checkpoint genes (Yang et al., 2019; Ma et al., 2020).

### Building the immune Landscape

The reduce dimension function of the “Monocle” package with a Gaussian distribution was used to perform dimensionality reduction analysis based on graph learning to visualise the distribution of immune subtypes among individual patients, and the maximum number of components was set at 2. Visualisation of the immune landscape was achieved using the functional plot cell trajectories of the immune subtypes, which were characterised by different colours.

## Results

### Identification of Putative Mesothelioma Antigens

**FIGURE 1:**
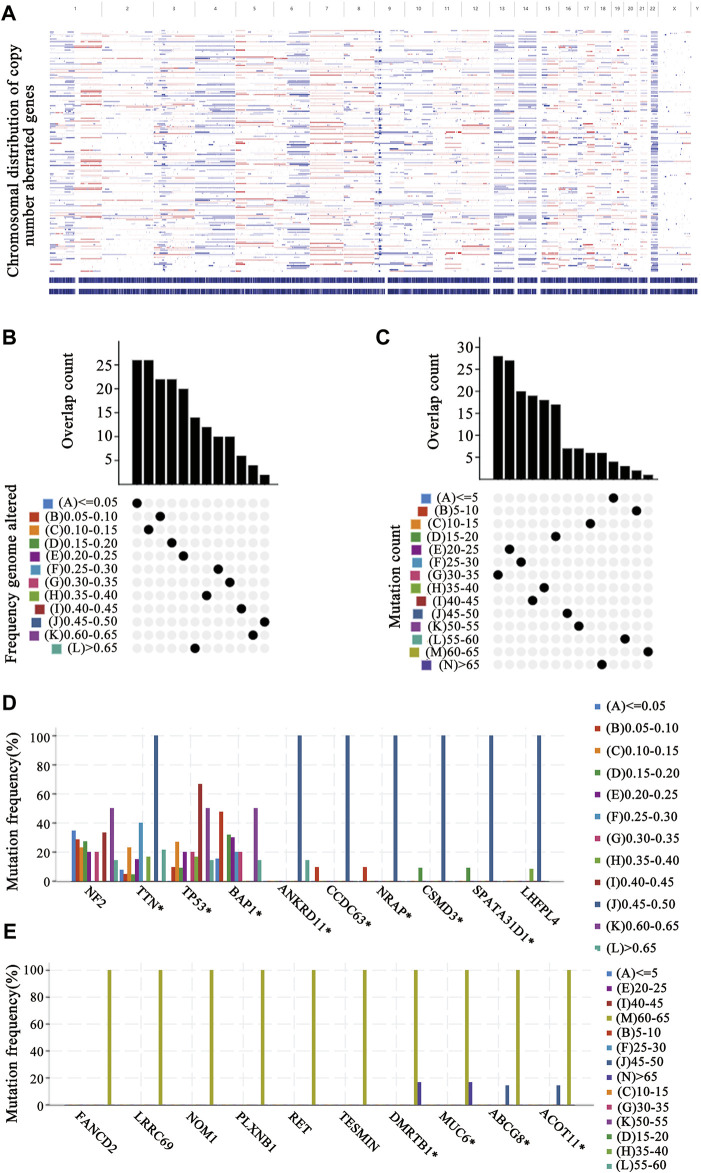
Identification of tumor antigen candidates **(A)** The distribution map of copy number aberrant genes on the chromosomes **(B–E)** Identification of tumor-specific antigen candidates. The overlapping distribution of mutated genes in fraction genome altered groups **(B)** and mutation count groups **(C)**. The highest frequency genes in altered genome fraction groups **(D)** and mutation count groups **(E)**.

To identify putative tumor antigens in mesothelioma, amplified and mutated genes were screened. By estimating the fraction of the genome alteration and the mutational load in individual samples, we identified 7,162 genes with increased copy number, 3,077 of which presented mutations (Figure 1A). These genomic alterations appeared with low frequency in most mesothelioma patients (Figures 1B,C), which suggests that mesothelioma exhibits a low immunogenic capacity. Further mutation analyses revealed that TTN, Tp53, BAP1, ANKRD11, CCD63, NRAP, CSMD, and SPATA31D1 were the most frequently mutated genes in terms of the fraction of the genome altered (Figure 1D), whereas DMRTB1, MUC6, ABCG8, and ACOT11 were the most frequently mutated genes based on mutation counts (Figure 1E). Overall, a total of 1,190 amplified and mutated genes were identified in mesothelioma.

### Identification of Prognosis- and APC-Related Tumor Antigens

**FIGURE 2:**
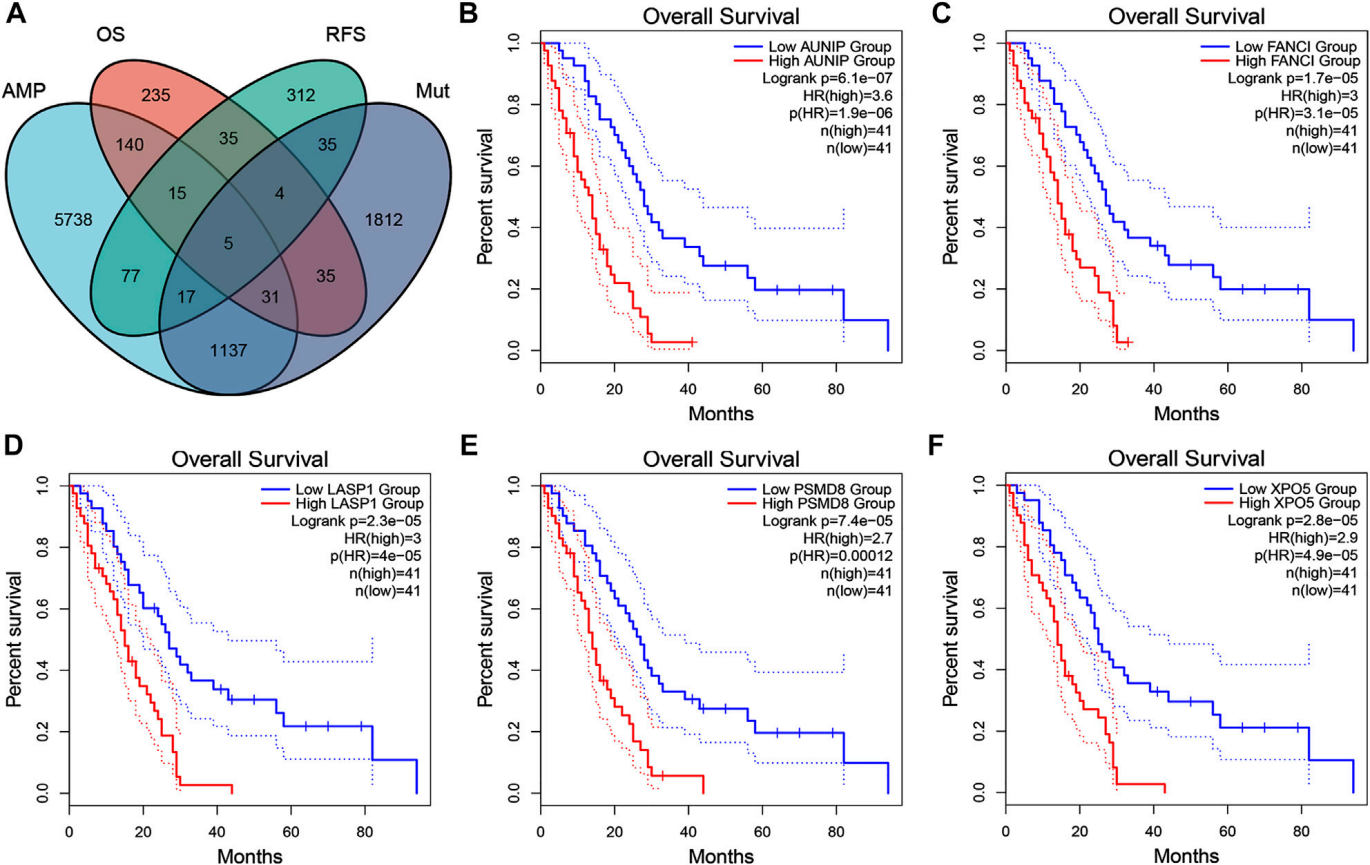
Identification of tumor antigen candidates related to mesothelioma prognosis **(A)** A total of five tumor antigen candidates with amplification and mutation characteristics, significantly associated with overall survival (OS) and progression-free survival (RFS) **(B–F)** Kaplan-Meier OS curves based on the expression levels of AUNIP **(B)**, FANCI **(C)**, LASP2 **(D)**, PSMD8 **(E)**, and XPO5 **(F)**, respectively.

**FIGURE 3:**
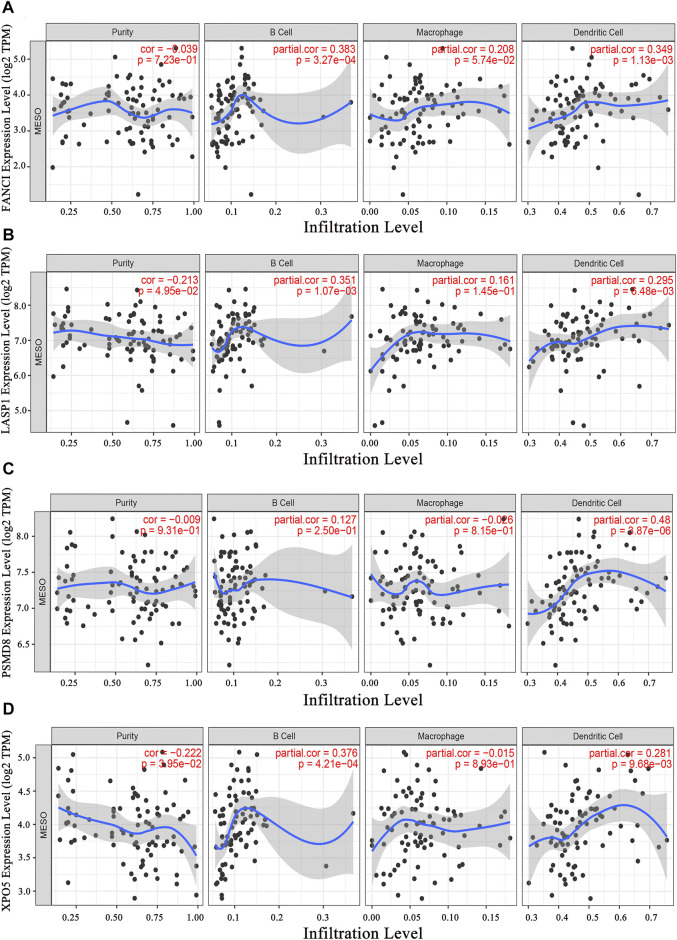
Identification of tumor antigen candidates related to antigen-presenting cells (APCs). The relationship of the expression of FANCI **(A)**, LASP1 **(B)**, PSMD8 **(C)**, and XPO5 **(D)** with the immune cell infiltration (B cells, macrophages, and dendritic cells).

Next, tumor antigens associated with prognosis were identified from the aforementioned genes as potential candidates for mRNA vaccine development. A total of 36 genes were significantly correlated with the OS of mesothelioma patients, five of which were associated with RFS (Figure 2A). A Kaplan-Meier survival analysis revealed that high expressions of AUNIP (Figure 2B), FANCI (Figure 2C), LASP1 (Figure 2D), PSMD8 (Figure 2E), and XPO5 (Figure 2F) were clearly associated with poor OS and RFS, suggesting that the five candidate genes play important roles in the tumorigenesis and progression of mesothelioma. Importantly, the expressions of FANCI and LASP1 were positively correlated with infiltration by B cells, macrophages, and dendritic cells (DCs) (Figures 3A,B). Furthermore, overexpression of PSMD8 and XPO5 genes was associated with increased infiltration of B cells and DCs (Figures 3C,D). There results suggest that the screened tumour antigens may be processed by APCs and the presented to T cells, which then interact with B cells to trigger an adaptive immune response. Validating this speculation will require further research. Taken together, five tumor antigens were validated as promising candidates for anti-mesothelioma mRNA vaccine development.

### Identification of Potential immune Subtypes

**FIGURE 4:**
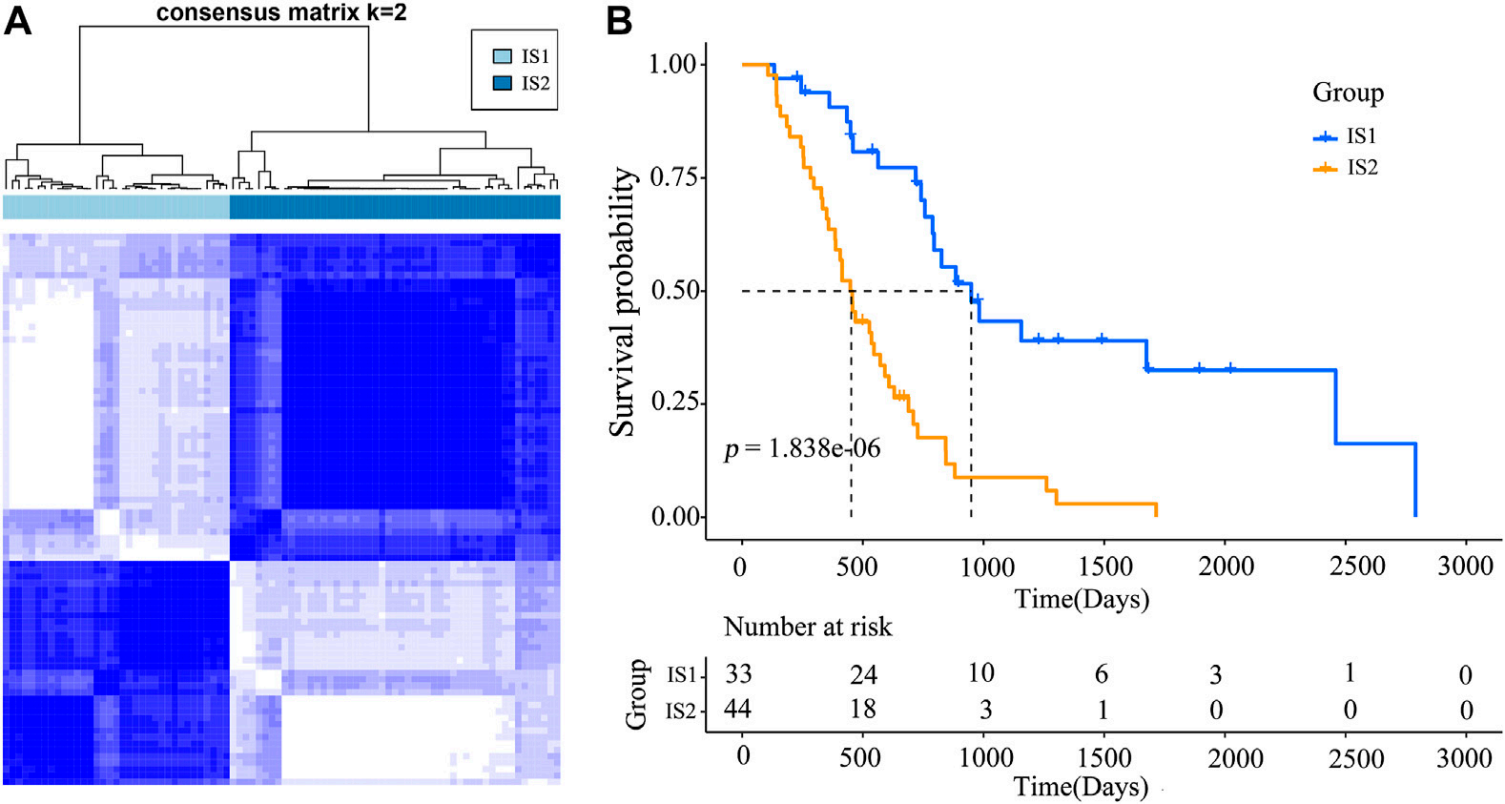
Immunophenotyping for mesothelioma **(A)** Consensus clustering analysis of samples on the basis of the expression of immune-related genes **(B)** Kaplan-Meier curves for the two immune subtypes.

Immunophenotyping characterizes the status of the tumor immune microenvironment, thus immunotyping may be useful for stratifying patients for mRNA vaccine therapy. Based on the expression of 2,108 IRGs in 86 samples from the TCGA database (gene list was shown in Supplymentary Table S1), two immune subtypes were identified (designated IS1 and IS2; Figure 4A) using a consensus clustering method. Notably, the two immune subtypes had remarkably different survival probabilities in that IS1 exhibited a superior prognosis than IS2 (TCGA cohort; Figure 4B), highlighting the prognostic significance of immunotyping.

### Correlation of immune Subtypes With Tumor Mutational Burden and Mutated Genes

**FIGURE 5:**
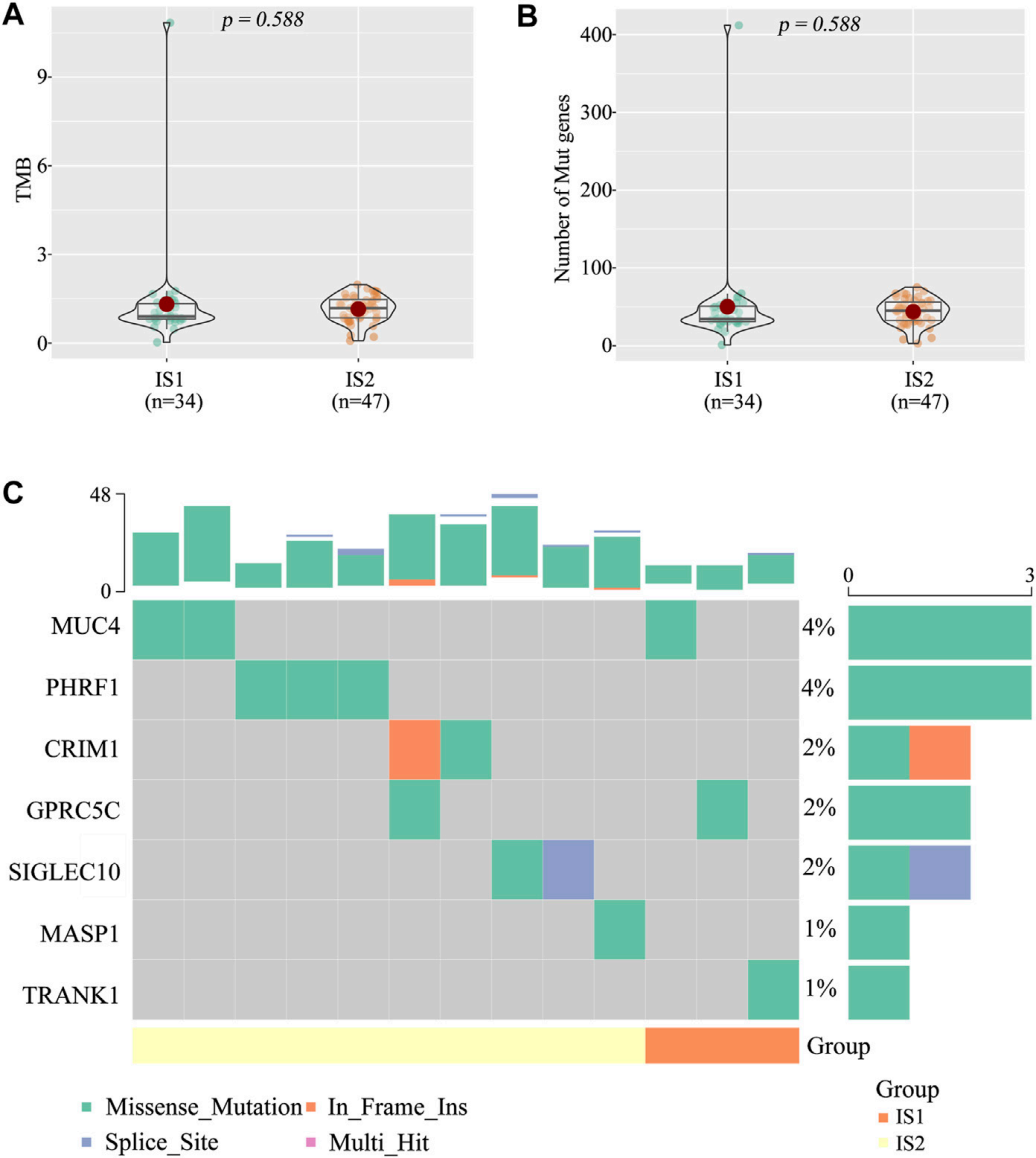
The tumor mutational burden (TMB) between the two immune subtypes **(A)** TMB **(B)** Number of gene mutations **(C)** Seven high mutation genes in the two immune subtypes.

High tumor mutational burden (TMB) and mutation frequency are associated with anti-tumor immunity and immunotherapeutic efficacy (Rooney et al., 2015). Therefore, we analyzed TMB and mutational status of the two immune subtypes using the mutect2-processed mutation dataset (TGCA cohort). Unfortunately, no notable discrepancies with respect to TMB (Figure 5A) and the number of mutated genes (Figure 5B) were observed between the two immune subtypes (IS1 and IS2). The landscape of seven IRGs with the most frequent genomic alterations was shown in Figure 5C. These results suggest that the number of mutated gene-encoded antigens was not different between the two immune subtypes.

### Relationship Between immune Subtypes and Immunomodulators

**FIGURE 6:**
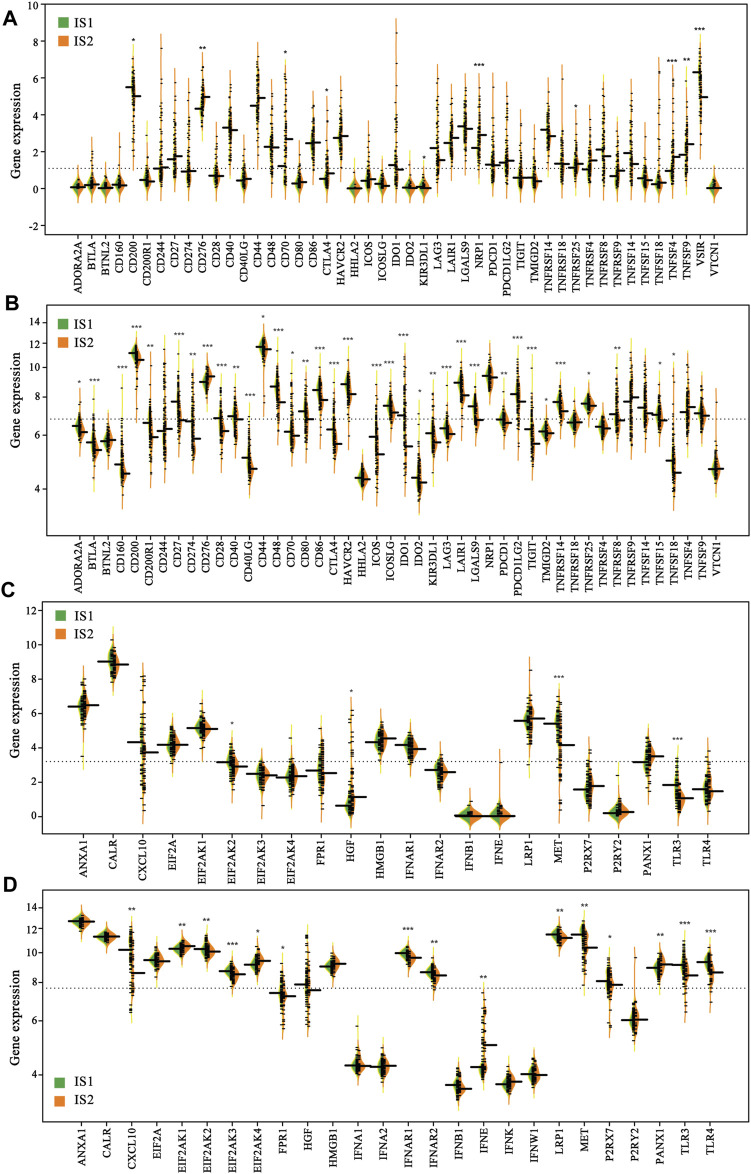
Difference in the expression of immune checkpoints (ICPs) and immunogenic cell death (ICD) modulators of the two immune subtypes **(A)** ICPs (TCGA cohort) **(B)** ICPs (GEO cohort) **(C)** ICPs (TCGA cohort) **(D)** ICPs (GEO cohort) (*p < 0.01, **p < 0.001, and, ***p < 0.0001).

Considering the significant role of immune checkpoints (ICPs) and immunogenic cell death (ICD) modulators in anti-tumor immunity and vaccine therapy, their expression levels in the IS1 and IS2 subtypes were evaluated. A total of 47 genes associated with ICPs were examined separately in two cohorts, of which 10 (21.3%) differentially expressed ICPs were in the TCGA cohort (Figure 6A) and 35 (74.5%) differently expressed ICPs were in the GEO cohort (Figure 6B). For the IS2 subtype, the vast majority of differentially expressed genes, including CD276, CD70, CTLA4, NRP1, TNFRSF25, TNFSF4, TNFSF9, were markedly increased in the TCGA cohort. Of note, the overall expression levels of the ICP genes in the TCGA cohort were lower than those in the GEO cohort. Twenty-two ICD genes were identified in the TCGA cohort, of which 4 (18.2%) were differentially expressed between the two subtypes (Figure 6C). Similarly, 26 ICD genes were detected in the GEO cohort, of which 15 (57.7%) exhibited significant differences between the IS1 and IS2 subtypes (Figure 6D). In the IS1 mesothelioma patients, the expressions of EIF2AK2, MET, and TLR3 were notably increased in the TCGA cohort, whereas CLCX10, EIF2AK2, EIF2AK3, FPR1, IFNAR1, IFNAR2, LRP1, MET, P2RX7, TLR3, and TLR4 were significantly overexpressed in the GEO cohort. Collectively, immunotyping was directly associated with the expression of the ICP and ICD modulators, and so acted as a biomarker for mRNA vaccine, which may be aid in identifying patients suitable for mRNA vaccination. mRNA vaccines may be less effective in patients with high ICPs expression and more effective in patients with upregulated ICD modulators.

### Tumor immune Microenvironment Features of the Immune Subtypes

**FIGURE 7:**
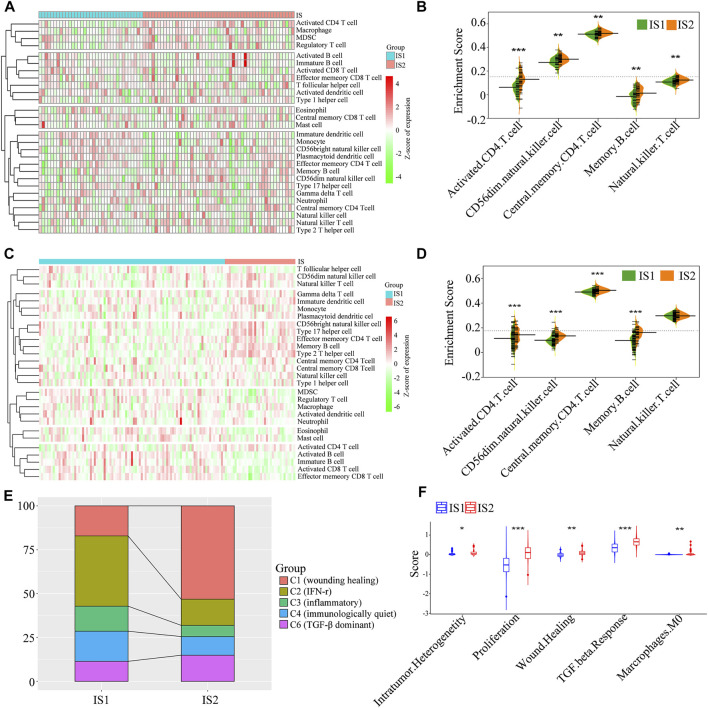
The cellular and molecular features of the two immune subtypes. The difference in the enrichment scores of immune cells between the two immune subtypes from the TCGA cohort **(A,B)** and GEO cohort **(C,D) (E)** The distribution of five pan-cancer immune subtypes in the mesothelioma immune subtypes **(F)** The difference in the enrichment scores of immune signatures between the two immune subtypes (*p < 0.01, **p < 0.001, and, ***p < 0.0001).

Because the tumor immune status largely determines the effectiveness of mRNA vaccines, the tumor immune microenvironment characteristics of the two immune subtypes were analyzed by estimating the scores of 28 infiltrating immune cells. A marked difference in composition of the infiltrating immune cells was observed between the IS1 and IS2 subtypes (Figure 7A). Notably, IS2 exhibited a rich infiltration of immune cells, including activated CD4^+^ T cells, CD56dim natural killer cells, central memory CD4^+^ T cells, and memory B cells in the TCGA cohorts (Figures 7A,B), similar to that of the GEO cohorts (Figures 7C,D). Therefore, IS2 was characterized as an immune “hot” phenotype, whereas IS1 showed an immune “cold” phenotype characterized by a lack of immune cell infiltration.

To validate the reliability of mesothelioma immunotyping, the relationship of the two immune subtypes and previously reported six immune categories (C1–C6) was determined (Thorsson et al., 2018). We found that five categories (C1, C2, C3, C4, and C6) exhibited distinct distribution and composition differences between the IS1 and IS2 immune subtypes (Figure 7E). For example, C2 (IFN-r), C3 (inflammatory), and C4 (immunologically quiet) were associated with IS1, whereas C1 (wounding healing), and C6 (TGF-β dominant) primarily gathered in IS2. These findings indicate that the tumor immune microenvironment of mesothelioma was significantly different compared with that of other tumor types, which may provide an additional supplement to previous studies. Subsequently, a correlation analysis between the two immune subtypes and 56 previously defined immune-related molecular characteristics revealed five significantly associated molecular signatures. As shown in Figure 7F, IS2 exhibited higher enrichment scores for intratumoral heterogeneity, proliferation, wound healing, and TGF-β response. IS2 showed enhanced immune infiltration and elevated scores for stromal fraction and TGF-β response, indicative of an immune “hot” and immunosuppressive phenotype, whereas lower immune cell infiltration scores and the TGF-β response signatures in IS1 suggest an immune “cold” phenotype, which confirmed the cell signature results. Taken together, immunotyping may reflect the tumor immune status and facilitate the stratification of patients for mRNA vaccination. The vaccine may enhance immune cell infiltration in patients with an immune “cold” IS1 tumor.

### Immunogenomic Landscape of Mesothelioma

**FIGURE 8:**
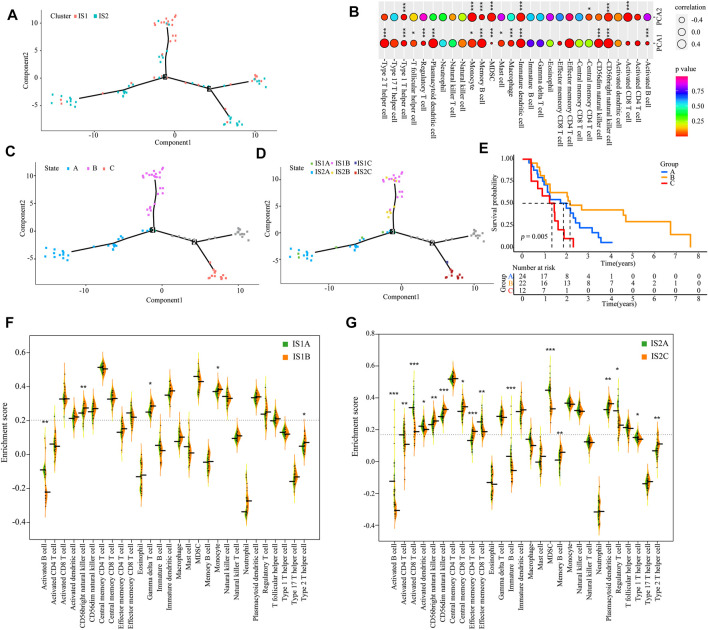
Immune landscape of mesothelioma **(A)** Immune landscape of mesothelioma **(B)** Correlation between PCA1/2 and immune modules. The immune landscape of patients from three extremely distributed locations **(C)**, and their Kaplan-Meier curves **(E) (D)** Further stratification of IS1 and IS2 based on their location on the immune landscape **(F,G)** The difference in the enrichment scores of immune signatures between the subsets. IS1A vs. IS1B **(F)**. IS2A vs. IS2B **(G)** (*p < 0.01, **p < 0.001, and, ***p < 0.0001).

According to the expression of IRGs, we generated an immunogenomic landscape of mesothelioma for visualizing the immune distribution of each patient, and identifying the population suitable for mRNA vaccine therapy from the IS1 subtype. A remarkably opposite immune landscape distribution was observed between the two immune subtypes (Figure 8A). The horizontal coordinate was closely associated with multiple types of immune cells. Type 2 helper T cell, plasmacytoid DCs, memory B cells, immature DCs, CD56^dim^ natural killer cells, and CD56^bright^ natural killer cells exhibited the highest positive correlation, whereas the vertical axis was positively associated with monocytes, myeloid-derived suppressor cells (MDSCs), mature DCs, CD56^bright^ nature killer cells, and activated CD8^+^ T cells (Figure 8B). Moreover, extremely distributed patients (gray data points are excluded) based on the immune landscape were obtained for subsequent analysis. The results indicate that patients in the B group had a higher survival rate (Figures 8C–E). Based on the location of the two immune subtypes, IS1 and IS2 patients could be each further stratified into three subgroups (Figure 8D), and the abundance of the infiltrating immune cells was markedly different between subgroups. For example, IS1A exhibited lower enrichment scores for CD56^bright^ natural killer cells, γδ T cells, monocytes, and type 2 helper T cells compared with the IS1B subgroup (Figure 8F), whereas IS2C displayed lower numbers of activated CD4^+^ T cells, activated CD8^+^ T cells, effector memory CD8^+^ T cells, regulatory T cells, MDSCs, and type 1 T helper cells compared with the IS2A subgroup (Figure 8G). Hence, the mRNA vaccination for mesothelioma patients may be more effective for IS1B patients. Taken together, immune subtype-based immune landscape can be used to ascertain the immune components of each mesothelioma patient and to predict clinical outcome. This is conducive to stratifying suitable patients for individualized treatment with mRNA vaccines.

### Identification of Co-expression Modules and Hub immune Genes in Mesothelioma

**FIGURE 9:**
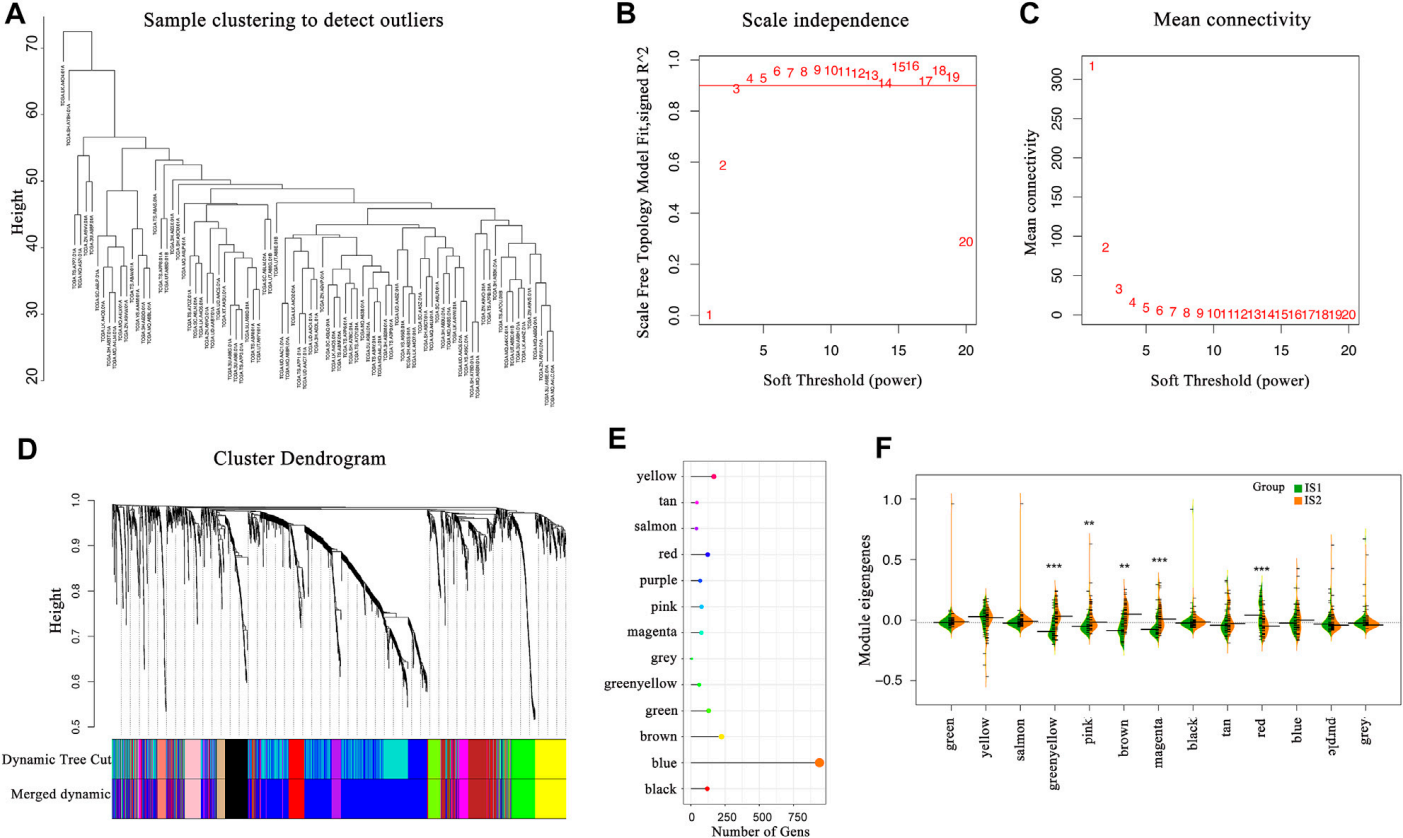
Identification of immune-related gene co-expression modules **(A–D)** Gene co-expression network analysis on the basis of immune-related genes **(E)** The number of genes in the different modules **(F)** Differential distribution of feature vectors of each module in the two immune subtypes.

**FIGURE 10:**
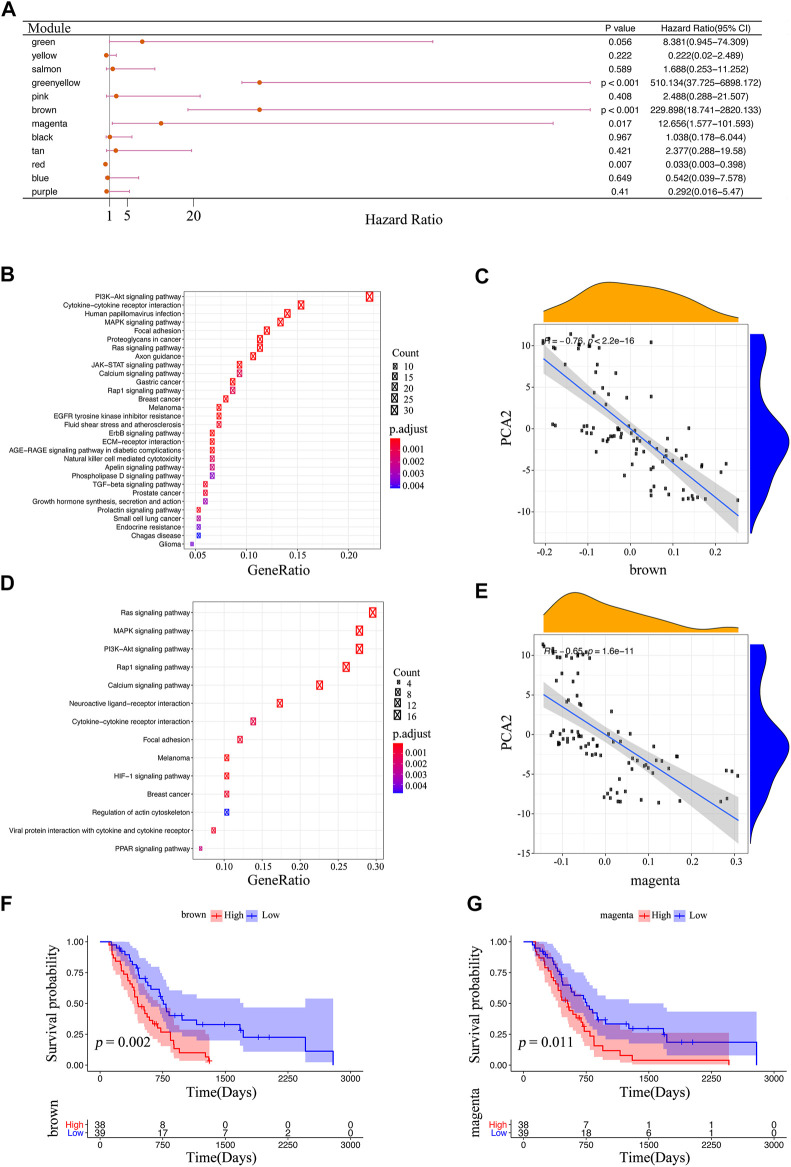
Identification of immune core genes **(A)** Survival analysis of eleven modules. The enrichment results of KEGG in the brown module **(B)** and the magenta module **(D) (C,E)** The association between the feature vector of the modules and PCA2 in the immune landscape; the brown module **(C)**, and the magenta module **(E) (F,G)** Kaplan-Meier curves of the brown module **(F)**, and magenta module **(G)**.

The co-expression modules of immune-related genes were applied to cluster IRGs, whose expression markedly affects the efficacy of mRNA vaccines. WGCNA was deployed to classify the IRGs for building co-expression modules (Figures 9A–C). A total of twelve gene co-expression modules were acquired (Figure 9D), whereas the genes in the gray module were not clustered with the rest of modules (Figure 9E). Next, we evaluated the distribution of two immune subtypes among these module eigengenes and found that IS1 eigengenes were evidently lower in greenyellow, pink, brown, and magenta modules (Figure 9F). Besides, the prognostic correlation analysis demonstrated that the greenyellow, brown, and magenta modules were significantly correlated with the survival of mesothelioma patients (Figure 10A). Subsequent functional enrichment analysis revealed that genes involved in the P13K-Akt signaling and cytokine-cytokine receptor interaction were enriched in the brown module (Figure 10B), whereas the genes involved in the Ras, MAPK, and P13K-Akt signaling pathways were enriched in the magenta module (Figure 10D). Interestingly, both brown and magenta modules were significantly negatively associated with component 2 of the immunogenomic landscape (Figures 10C,E). Consistently, patients with low expression scores of genes that were clustered into brown (Figure 10F) and magenta (Figure 10G) modules had superior outcomes compared with those with higher scores. Therefore, the mRNA vaccination strategy may be suitable for mesothelioma patients with low expressing genes clustered in the brown and magenta modules. Finally, two hub IRGs, TIE1, and CLEC1A, in the magenta module with a correlation >90% relevance were identified. They could be used as potential biomarkers for predicting survival probability and determining suitable mesothelioma patients for mRNA vaccination.

## Discussion

Malignant mesothelioma is a rare and aggressive solid tumor with no known cure (Li et al., 2019). Current immunotherapies have completely changed oncology from a therapeutic perspective, while its effectiveness in mesothelioma remains unclear (Ribas and Wolchok, 2018). Vectors are one of the flexible means of vaccine delivery. Although viral vector-based cancer vaccines have the advantage that the entire tumor antigen gene or a portion of the gene can be inserted and several genes can be inserted into the vector, there are several challenges to wide clinical application of these vaccines due to the complex manufacturing and the immune responses against components of the viral vector backbone (Larocca and Schlom, 2011). Notably, comparing with viral vector-based cancer vaccines, the delivery vehicles of mRNA vaccines transfect immune cells without inducing toxicity or unwanted immunogenicity, such as lipid-based nanoparticles, and polyplexes and polymeric nanoparticles (Sahin and Türeci, 2018). Compared to mRNA vaccines, peptide vaccines have several disadvantages that limit their clinical potential. For example, high variability in physicochemical properties of individual peptides complicates fabrication. The second challenge is unrelated immune responses against artificial epitopes generated by peptide degradation in the extracellular space (Larocca and Schlom, 2011; Sahin and Türeci, 2018). The mRNA-based vaccine is well-suited for targeting tumor-specific antigens (Huang et al., 2021a; Huang et al., 2021b). It has achieved significant clinical effects and may represent a new avenue for cancer immunotherapy, however, the effects of mRNA vaccines on patients with mesothelioma have not been determined.

In this study, we identified five tumor antigens (AUNIP, FANCI, LASP1, PSMD8, and XPO5) that were considered promising candidates as mRNA-based vaccines. Their overexpressions were associated with adverse prognosis and RFS together with elevated APCs and B cell infiltration, which may play important functional roles for the occurrence and development of mesothelioma. Thus, targeting these antigens may promote APC and B cell infiltration and induce a host immune response. As expected, previous reports have also indicated that they have the potential of becoming targets for the development of mRNA vaccines. AUNIP, also known as AIBp and C1orf135, plays an important role in DNA damage repair and the cell cycle. AUNIP expression is associated with the tumor microenvironment and immune infiltration in oral squamous cell carcinoma, hepatocellular carcinoma, and lung adenocarcinoma (Yang et al., 2019; Ma et al., 2020). Thus, it is considered a candidate diagnostic and prognostic biomarker for these cancers. FANCI is a key protein in DNA repair and ribosome biogenesis and cooperates with IMPDH2 to promote cell proliferation of (lung adenocarcinoma) LUAD (Zheng and Li, 2020). LASP1 is an actin-binding protein and can enhance tumor growth and metastasis in multiple cancers, including nasopharyngeal carcinoma, colorectal cancer, triple-negative breast cancer, and pancreatic ductal adenocarcinoma (Hu et al., 2017; Butt and Raman, 2018). PSMD8 was found to be overexpressed or mutated in breast cancer and diffuse large B cell lymphoma (Yuan et al., 2021). XPO5 is responsible for the nuclear export of pre-miRNA into the cytoplasm and is correlated with tumorigenesis and prognosis in various cancers (Patrão et al., 2018; Clancy et al., 2019).

Because the benefits of survival and therapeutic responses of patients subjected to mRNA vaccine-based cancer immunotherapy remain limited to a small population, stratifying patients to obtain better insight for the optimal use of tumor immunotherapy is an effective strategy. According to the expression profiles of IRGs, patients with mesothelioma may be divided into two immune subtypes. They exhibited significantly different cellular, molecular and clinical characteristics. A previous study identified six immune subtypes by performing an immunogenomics analyses (Thorsson et al., 2018). Patients with C1, C2, and C3 were correlated with excellent prognosis, C4 and C6 were associated with poor prognosis. In the present study, mesothelioma patients were classified into five subtypes (C1, C2, C3, C4, and C6). C2 and C3 were mainly clustered into the IS1 group, and C6 was associated with the IS2 group. These results were consistent with a better survival rate for IS1, and a worse survival for IS2. Moreover, IS1 eigengenes were significantly higher in greenyellow, brown, and magenta modules characterized by an unfavorable prognosis, which also confirmed the prognostic classification (IS1 and IS2). Taken together, these findings demonstrate the reliability of immunotyping and augment the previous classification.

Considering that the tumor immune status is a key factor influencing the effectiveness of mRNA vaccines, the tumor immune microenvironment characteristics of two immune subtypes were evaluated. IS1 exhibited an immune “cold” phenotype characterized by a lack of immune cell infiltration, indicative of a non-inflammatory TME (Huang et al., 2021a; Huang et al., 2021b). In contrast, IS2 displayed TME characteristics that were opposite to those of the immune “cold” phenotype, and were characterized by an enhanced immune cell infiltration and elevated scores for TGF-β response, indicative of an immune “hot” and immunosuppressive phenotype (Huang et al., 2021a; Huang et al., 2021b). These two immune subtypes could represent the distinct potential mechanisms that modulate tumor immune escape, corresponding to different treatment strategies. Recent studies suggest that patients with mesothelioma undergoing immunotherapy (e.g., T cell therapies) or chemotherapy (e.g., gemcitabine) showed enhanced tumor immune cell infiltration (e.g., CD4 + T helper cells and CD8 + T cells) and prolonged survival (de Gooijer et al., 2020; Gray and Mutti, 2020). Therefore, immunotherapy using mRNA vaccines targeting previously recognized tumor antigens (AUNIP, FANCI, LASP1, PSMD8, and XPO5) could induce immune cell infiltration to reinvigorate the ISI patient’s immune system. Furthermore, the molecular signatures of IS1 (e.g., low scores of TGF-β response, low expression of ICPs, and overexpression of ICD genes) indicate that these patients are suitable for mRNA vaccination therapies. Suzuki et al. revealed that blocking TGF-β signal transduction significantly enhanced the anti-tumor response of CD8^+^ T cells, and ultimately inhibited the growth of malignant mesothelioma (Suzuki et al., 2007). Combaz-Lair et al. found that TLR3 could restore the anti-tumor immune response in patients with mesothelioma (Combaz-Lair et al., 2016). EIF2AK2 is an interferon-inducible, double-stranded RNA protein kinase and its activation can suppress the metastatic capabilities of several cancer cell types (Garcia-Ortega et al., 2017). The MET gene encodes a receptor tyrosine kinase, c-MET, that binds to hepatocyte growth factor and dysregulation of MET signaling is implicated in cell migration, proliferation, and angiogenesis (Guo et al., 2020). The hot tumor (IS2) corresponded to a more complex TME. Inflammatory cytokines, such as, interleukin, tumor necrosis factor and TGF-β, play key roles in tumor progression and promotion of early metastasis (Han et al., 2020). Although IS2 exhibited increased immune cell infiltration, the survival of IS2 patients was worse than that of IS1. Thus, the key factor in determining the prognosis may be the immunosuppressive environment or the stimulation environment dominating. In the immune-suppressive environment, tumors exhibited the high expressions of TGF-β and ICPs, as well as poorly immunogenic of cancer cell death. Previous studies demonstrated that the activated TGF-β signaling pathways may suppress immune function by preventing lymphocytes from entering the tumor parenchyma, whereas specific molecular inhibitors that target TGF-β restored anti-tumor immunity by remodeling the tumor immune microenvironment (Travis and Sheppard, 2014; Larson et al., 2020). Thus, patients exhibiting the IS2 subtype may benefit from combination therapy of ICP inhibitor drugs and TGF-β blockers.

The complex tumor immune microenvironment implies that there is heterogeneity within the same immune subtype and between individual patients. Compared with IS1A, patients with IS1B showed a relative increase in tumor-infiltrating T cells. This suggests that combination therapy with mRNA vaccine and ICP inhibitors may alter the tumor microenvironment in these patients to a state that is more conducive to successful treatment by promoting immune cell infiltration and enhancing an anti-tumor T cell response. We also identified that IS2 patients may be unsuitable for RNA vaccines compared with IS1, however, mRNA vaccines could be relatively feasible for IS2C patients among the IS2A, IS2B, and IS2C subtypes. Of note, it is important to integrate the results of the two immune subtypes and the immune landscape for mesothelioma. Furthermore, to shrink the immune components used to develop personalized therapies based on mRNA vaccines, the co-expression modules of immune gene were constructed. The two co-expression modules with hub IRGs (TIE1 and CLEC1A) were negatively correlated with component 2 of the immune landscape, implying that patients with low expression of the two genes could be suitable for mRNA vaccines. This paper has some limitations that further experimental verification are needed to validate these findings, including mRNA vaccine synthesis and purification, and murine models with mesothelioma.

## Conclusion

AUNIP, FANCI, LASP1, PSMD8, and XPO5 are putative tumor antigens for mRNA vaccine development. Patients with the IS1 subtype could be more suitable for mRNA vaccination. This study provides a theoretical foundation for developing effective tumor vaccine therapy for mesothelioma patients.

## Data Availability

The datasets presented in this study can be found in online repositories. The names of the repository/repositories and accession number(s) can be found in the article/Supplementary Material.

## Abbreviations

ICP, Immune checkpoint; DC, Dendritic cell; iPSC, Induced pluripotent stem cells; FPKM, Fragments Per Kilobase of exon model per Million mapped fragments; RFS, Relapse-free survival; OS, Overall survival; GO, Gene oncology; TSAs, tumor-specific antigens; IRGs, Immune-related genes; APC, Antigen-presenting cell; IS, Immune subtypes; TCGA, The Cancer Genome Atlas; GEO, Gene expression omnibus; GEPIA, Gene Expression Profiling Interactive Analysis; cBioPortal, The cBio Cancer Genomics Portal; TIMER, The Tumour Immune Estimation Resource; PAM, Partition around medoids; SSGSEA, Single-sample Gene Set Enrichment Analysis; TMB, Tumor mutational burden; ICD, Immunogenic cell death. LUAD, lung adenocarcinoma.
