# Supplementary figures and images for: Identification of Potential Antigens for Developing mRNA Vaccine for Immunologically Cold Mesothelioma
Source: Front Cell Dev Biol. 2022 Jul 1;10:879278. doi: 10.3389/fcell.2022.879278 (PMC9284534; doi:10.3389/fcell.2022.879278)

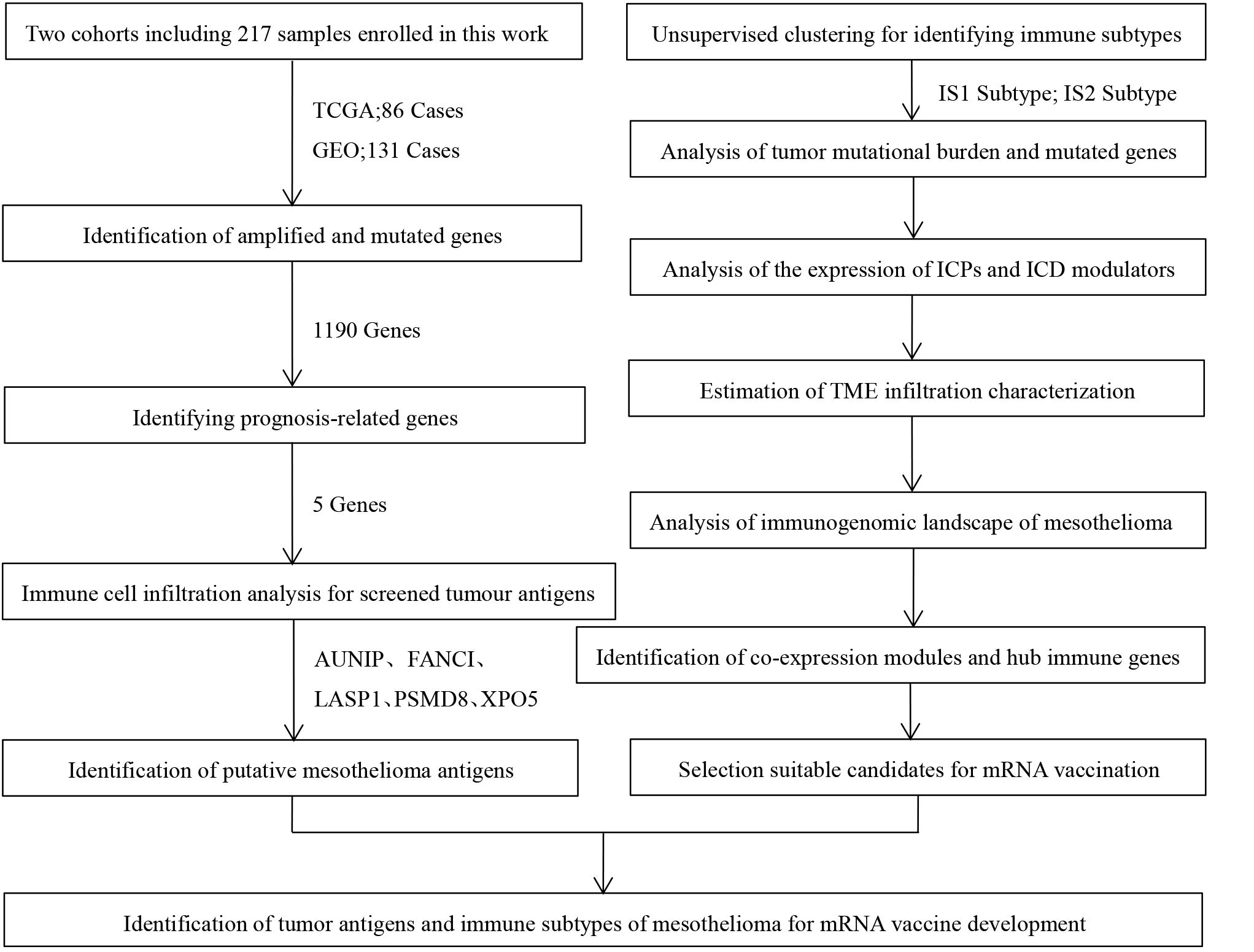

Supplement: Supplementary file 2 [file Image1.TIF]
